# A Bioengineered Glycosyl Hydrolase Effectively Disrupts *Campylobacter jejuni* Biofilms

**DOI:** 10.3390/foods15162849

**Published:** 2026-08-15

**Authors:** Yiping He, Chin-Yi Chen, Gretchen Dykes, Heather Koppenhöfer, Joseph Capobianco, Kevin Lynn, Bryan Berger

**Affiliations:** 1 Characterization and Interventions for Foodborne Pathogens Research Unit, Eastern Regional Research Center, Agricultural Research Service, United States Department of Agriculture, 600 East Mermaid Lane, Wyndmoor, PA 19038, USA; chin-yi.chen@usda.gov (C.-Y.C.);; 2 Department of Chemical Engineering, University of Virginia, Charlottesville, VA 22904, USA

**Keywords:** *Campylobacter*, biofilms, CAase, glycosyl hydrolase, foodborne pathogen, food safety

## Abstract

Biofilms contribute significantly to the persistence and transmission of *Campylobacter jejuni*, yet enzymatic strategies for disrupting these structures remain underexplored. In this study, we evaluated the activity of a glycosyl hydrolase, CAase, against both planktonic and biofilm-associated *C. jejuni*. CAase showed no inhibitory effect on planktonic growth at concentrations up to 1 mg/mL. However, at 0.1 mg/mL, CAase reduced mature biofilm biomass by more than 93% in the wild-type strain and produced similar reductions in a *luxS* mutant, demonstrating substantial degradation of conserved glycan components within the extracellular polymeric substance (EPS). Scanning electron microscopy (SEM) imaging revealed substantial extracellular matrix loss, structural disruption, and altered cell morphology following CAase treatment. Rheological measurements demonstrated marked decreases in kinematic viscosity, further reflecting weakened biofilm cohesion. Together, these results indicate that CAase effectively disrupts multiple components of *C. jejuni* biofilm architecture despite lacking direct antimicrobial activity. This level of biofilm removal suggests potential utility in poultry-processing or surface-sanitation applications where targeted enzymatic disruption could enhance cleaning efficacy.

## 1. Introduction

*Campylobacter jejuni* is a leading cause of bacterial gastroenteritis worldwide and remains a significant challenge to food safety, particularly within poultry production and processing environments [1,2]. As a microaerobic, thermophilic organism, *C. jejuni* thrives in low-oxygen environments such as the avian gastrointestinal tract and can persist in protected microenvironments on food-contact surfaces and equipment. These physiological traits enable its survival in processing facilities despite its fragility under fully aerobic conditions [3,4,5]. Its low infectious dose and ability to contaminate carcasses, processing water, and equipment surfaces further contribute to its significance as a foodborne pathogen [6,7]. In addition, *Campylobacter* may enter dormancy and integrate into resident biofilms, making surface colonization and environmental persistence significant concerns. Therefore, effective strategies to limit biofilm formation and persistence are essential for reducing *Campylobacter*-associated illness.

Biofilm formation is a major contributor to the environmental resilience of *C. jejuni*. Biofilms enable attachment to stainless steel, plastic, rubber, and other industrial materials, protecting cells from desiccation, temperature fluctuations, and standard sanitation procedures [8,9,10,11]. This protection arises from the extracellular polymeric substance (EPS) matrix, which promotes cell aggregation, adhesion, and resistance to shear forces [12,13]. Unlike many bacteria with polysaccharide-dominated matrices, *C. jejuni* biofilms are comparatively protein-rich, contributing to greater mechanical stability, lower permeability, and reduced susceptibility to carbohydrate-targeted sanitizers [14]. Consistent with this, *C. jejuni* in mixed-culture biofilms shows reduced susceptibility to quaternary ammonia, peracetic acid, and peracetic acid/peroctanoic acid mixtures, with incomplete inactivation under these treatments [15]. Even so, *C. jejuni* EPS contains key glycan components, including capsular polysaccharides, glycoproteins, and LOS-associated carbohydrates, that support matrix cohesion and remain promising enzymatic targets [16,17].

Biofilm formation varies considerably among different *Campylobacter* strains, with *C. jejuni* 81-176, YH001, and YH027 forming robust monoculture biofilms [18]. Quorum sensing further modulates biofilm development via LuxS, which produces the interspecies signaling molecule autoinducer-2 (AI-2) [19]. *luxS* mutation disrupts AI-2 production and alters multiple biofilm-related phenotypes, including EPS composition, surface attachment, and matrix architecture. As a result, *luxS* mutants often produce biofilms with reduced biomass or modified structural properties [20,21]. These phenotypic differences make *luxS*-deficient strains valuable tools for evaluating whether biofilm-disrupting agents target conserved matrix components or vary in effectiveness across different EPS backgrounds.

Glycosyl hydrolases (GHs) have emerged as effective antibiofilm agents because they specifically cleave carbohydrate linkages within the EPS, targeting polysaccharide components essential for matrix integrity [22,23,24]. By hydrolyzing glycosidic bonds, GHs weaken the biofilm scaffold, enhance antimicrobial penetration, and facilitate detachment of sessile cells [25,26]. CAase, a bioengineered GH, disrupts EPS glycans and has been shown to remove mature biofilms of *Escherichia coli* O157:H7, *Salmonella enterica* serotype Typhimurium, and *Listeria monocytogenes*, achieving 17.4–34.6% greater removal than saline controls in crystal violet assays [27]. Despite its demonstrated efficacy across multiple foodborne pathogens, CAase has never been evaluated against *C. jejuni*. Given that *C. jejuni* produces a protein-rich yet glycan-containing EPS, it remains unclear whether glycan-cleaving enzymes can effectively disrupt its biofilms.

This study addresses this gap by evaluating CAase activity against *C. jejuni* 81-176 wild-type and *luxS*-mutant strains to determine whether glycan-directed disruption is effective across differences in EPS composition. Specifically, we assessed CAase effects on planktonic growth and mature biofilms and characterized structural and mechanical alterations using biomass quantification, scanning electron microscopy, and rheometry. By examining how CAase targets glycan components within a predominantly protein-rich matrix, this work provides critical insight into the feasibility and applied potential of glycan-cleaving enzymes for controlling persistent *C. jejuni* biofilms.

## 2. Materials and Methods

### 2.1. Minimal Inhibitory Concentration (MIC) Assay

Inhibition of *C. jejuni* growth by CAase was assessed using a broth microdilution assay as previously described [28]. The *C. jejuni* 81-176 strain was used because it is a well-characterized, highly virulent, and robust biofilm-forming strain, making it a standard and reliable model for evaluating CAase activity. Briefly, *C. jejuni* 81-176 was grown overnight in Mueller–Hinton (MH) broth (Becton Dickinson and Company, Franklin Lakes, NJ, USA), consisting of a standard dehydrated formulation containing beef extract, acid hydrolysate of casein, and starch, at 42 °C under microaerophilic conditions generated using a CampyPak gas pack (Becton Dickinson and Company), which produces an atmosphere of approximately 5% O_2_ and 10% CO_2_ (balance N_2_) in a sealed Nalgene Bio-Safe Carrier container (Rochester, NY, USA). The resulting culture was diluted in MH broth to approximately 10^4^ CFU/mL. An aliquot of this suspension was heat-killed at 99 °C for 10 min, cooled to room temperature, and used as the nonviable control.

Serial two-fold dilutions of CAase (0.001953125–1.0 mg/mL; 100 µL per well) were prepared in MH broth in sterile 96-well microtiter plates. An additional 100 µL of the bacterial suspension (~10^4^ CFU/mL) was added to each well, with column 11 receiving the heat-killed inoculum and all remaining wells receiving live cells. After incubating the plates at 42 °C for 48 h under microaerobic conditions, optical density at 600 nm was measured using a Safire 2 microplate reader (Tecan Group Ltd., Männedorf, Switzerland). Each CAase concentration was tested using four biological replicates, and growth inhibition in each treatment well was compared with that of the CAase-free control wells. Statistical significance of differences between treatment and control groups was evaluated using an all-pairs Tukey–Kramer test (α = 0.05) in JMP Pro (version 18.2, SAS Institute Inc., Cary, NC, USA).

### 2.2. Biofilm Sampling

Fresh colonies of *C. jejuni* 81-176 and its *luxS* deletion mutant [29] were resuspended in 2 mL of MH broth and incubated overnight at 42 °C under microaerophilic conditions. One milliliter of the overnight culture was then inoculated into 100 mL of MH broth and mixed thoroughly. From this inoculum, three different setups with distinct substrate type and surface area were used to develop biofilms: (1) six 2 mL aliquots were dispensed into 14 mL sterile polypropylene tubes (17 × 100 mm, Becton, Dickinson and Company) to evaluate biofilm formation in a small-volume, limited-surface-area environment; (2) six 5 mL aliquots were transferred into 10 mL polystyrene tissue culture tubes with a flat surface of 10 cm^2^ growth area and vent cap (Techno Plastic Products AG, Trasadingen, Canton Schaffhausen, Switzerland); and (3) six 5 mL aliquots were transferred into 40 µm nylon cell strainers (Becton, Dickinson and Company) positioned within a 6-well plate. These disposable mesh filters, designed to retain larger particulates, were used to provide a uniform, high-porosity surface that supports consistent biofilm attachment and enables efficient biomass recovery for downstream processing. Biofilms were allowed to develop for 5 days under microaerophilic conditions [18]. After incubation, biofilm biomass was harvested by filtering each sample through a 40 µm cell strainer fitted onto a 50 mL Falcon tube. Samples grown in the flat tubes were scraped with a silicon-blade cell scraper (Corning Life Sciences, Corning, NY, USA) to facilitate release of the biofilms.

### 2.3. Biofilms Quantification

Following CAase treatment, biofilms retained on the cell strainers were quantified using the crystal violet (CV) staining method as previously described [18]. Briefly, each biofilm sample was gently rinsed with 2 mL of MH broth, stained with 5 mL of 0.1% CV for 30 min, and washed three times with 5 mL of 18 MΩ water. The CV-stained biofilms were then solubilized by adding 6 mL of 100% ethanol and incubating at room temperature for 30 min with gentle shaking. Absorbance was measured in triplicate at 590 nm using a Cytation 5 plate reader (BioTek/Agilent Life Sciences, Winooski, VT, USA).

### 2.4. CAase Production

CAase was produced following the method of Mayton et al. [27]. Briefly, the enzyme, derived from a truncated *Salmonella* phage hydrolase (NCBI YP_004893855.1) and engineered with an N-terminal His_6_ tag, was expressed in *E. coli* BL21(DE3) under a T7 promoter (pET28) using IPTG induction and purified by Ni-NTA affinity chromatography. The purity of CAase was verified by SDS–PAGE, confirming successful purification of the final enzyme preparation. Enzyme concentration was determined by measuring absorbance at 280 nm, using a NanoDrop 1000 Spectrophotometer (Thermo Fisher Scientific, Waltham, MA, USA) and a calculated extinction coefficient of 121,990 M^−1^ cm^−1^ derived from its primary amino acid sequence.

### 2.5. CAase Treatment

Biofilm cells harvested on a cell strainer were rinsed with 2 mL of fresh MH broth and transferred to a 6-well plate. An aliquot of the culture filtrate was serially diluted in MH broth and enumerated using the 6 × 6 drop plate method [30] to quantify CFU in the planktonic fraction of the pre-treatment culture. For each biofilm growth setup, three biofilm samples in cell strainers were submerged in 5 mL of 0.1 mg/mL CAase. As untreated controls, three additional biofilms were submerged in 5 mL of MH broth alone. The 6-well plate containing the treated and untreated biofilms was incubated at 42 °C under microaerobic conditions generated with a CampyPak in an airtight container for 2 h.

### 2.6. Scanning Electron Microscope (SEM) Examination

Biofilms treated with or without 0.1 mg/mL CAase for 2 h under microaerobic conditions were collected by centrifugation (6000× *g*, 2 min) and applied directly to cleaned glass coverslips. Planktonic cells grown for 24 h at 42 °C under microaerobic conditions were included as a control. Samples were air-dried for 1 h at room temperature and fixed with 2.5% glutaraldehyde for 2 h. After rinsing with 0.1 M imidazole buffer, samples were dehydrated in 50%, 80%, and 100% ethanol (three changes per step) and dried by CO_2_ critical-point drying. Coverslips were mounted on SEM stubs and sputter-coated with a thin gold layer using a Scancoat Six sputter coater (BOC Edwards, Wilmington, MA, USA). SEM images were acquired at room temperature using a Quanta 200 FEG scanning electron microscope (FEI, Inc., Hillsboro, OR, USA) [28].

### 2.7. Viscosity Measurement of C. jejuni Biofilms

The kinematic viscosity of *C. jejuni* biofilms treated with or without 0.1 mg/mL CAase for 2 h under microaerobic conditions was measured using a calibrated glass kinematic viscometer tube (0.1 cSt/s constant flow rate; size 200; Thermo Fisher Scientific, Waltham, MA, USA) following the manufacturer’s instructions. After incubation, 5 mL of each biofilm sample was loaded into the viscometer tube. A vacuum pump was used to gently draw the sample up the capillary until the meniscus was slightly above the upper timing mark, after which the vacuum was released to allow the sample to flow downward under gravity. The efflux time, the time required for the meniscus to pass between the timing marks, was recorded at room temperature using video capture. Kinematic viscosity (ν in centistokes (cSt)) was calculated using the equation:

ν = t × C

where t is the efflux time (seconds), and C is the viscometer’s calibration constant (0.1 cSt/s). All measurements were performed on triplicate samples.

## 3. Results

### 3.1. Effect of CAase on C. jejuni Growth

**Figure 1:**
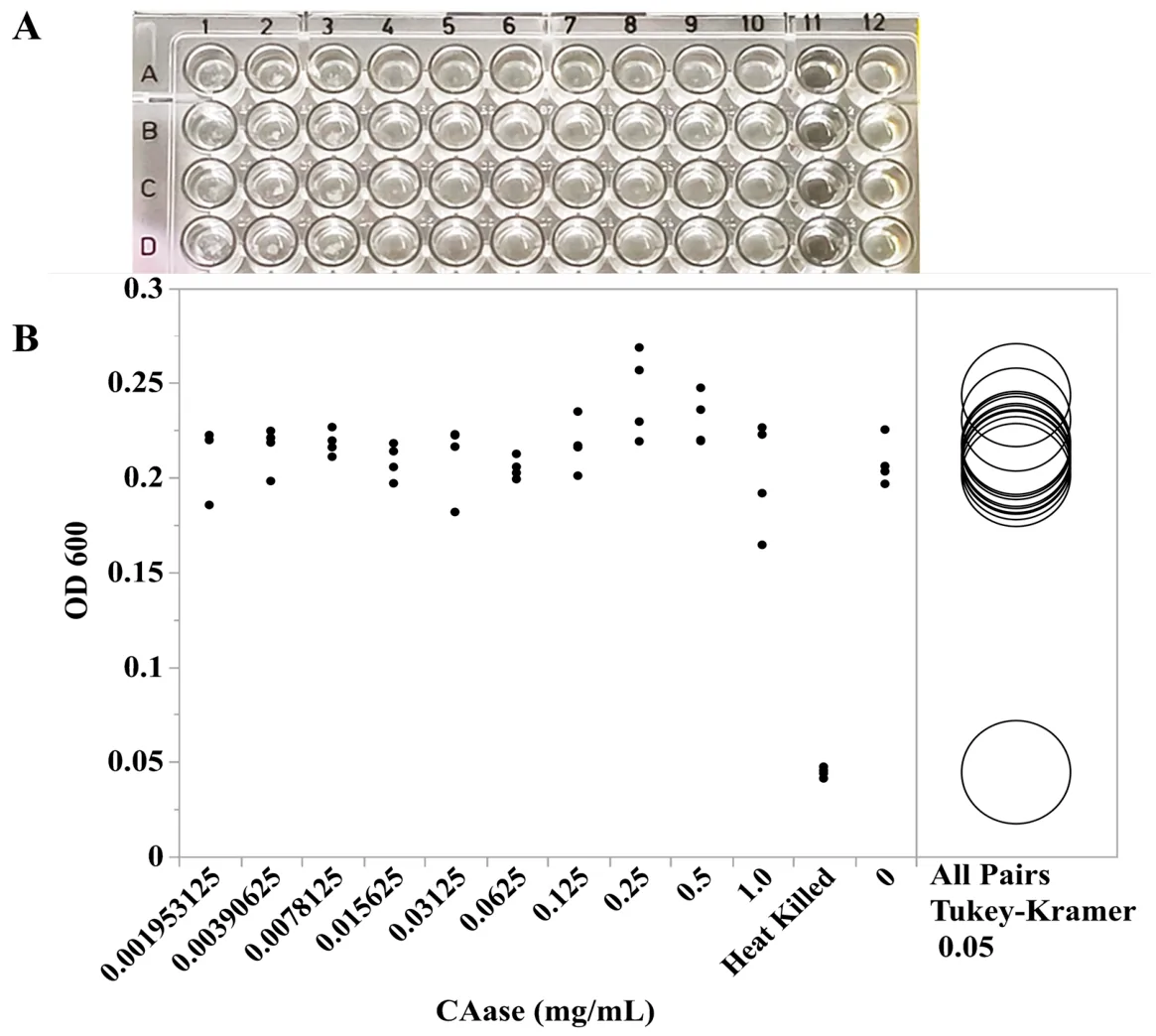
Effect of CAase on *C. jejuni* growth. Growth of *C. jejuni* 81-176 was evaluated across CAase concentrations ranging from 0.001953125 to 1.0 mg/mL. After 48 h of microaerobic incubation, cultures were photographed (**A**), showing CAase-treated wells (columns 1–10), a heat-killed control (column 11), and an untreated control (column 12). (**B**) Optical density (OD_600_) was measured following incubation. Data represent four biological replicates, and statistical comparisons were performed using the Tukey–Kramer test (α = 0.05).

Minimal inhibitory concentration (MIC) testing demonstrated that CAase did not inhibit the growth of *C. jejuni* 81-176 at concentrations up to 1 mg/mL (Figure 1). After 48 h of microaerobic incubation, OD_600_ values across the full CAase concentration range (0.001953125–1.0 mg/mL) were indistinguishable from the untreated control. Statistical analysis using the all-pairs Tukey–Kramer test (α = 0.05) confirmed that none of the CAase treatments resulted in a significant reduction in bacterial growth. As expected, only the heat-killed control showed no detectable growth (*p* ≤ 0.05). An independent trial conducted after 24 h of microaerobic incubation produced similar results. Collectively, these findings indicate that CAase lacks measurable antimicrobial activity against *C. jejuni* under the conditions tested, suggesting that any observed biological effects are likely due to mechanisms other than direct growth inhibition.

### 3.2. CAase-Mediated Disruption of C. jejuni Biofilms

The ability of CAase, a glycoside hydrolase that targets carbohydrate components of the EPS matrix, to disrupt biofilms was evaluated using *C. jejuni* 81-176 and its *luxS* mutant. Mature biofilms were exposed to 0.1 mg/mL CAase for 2 h under static, microaerobic conditions, and residual biomass was quantified using crystal violet staining (OD_590_). All experiments were performed in triplicate independent cultures.

**Figure 2:**
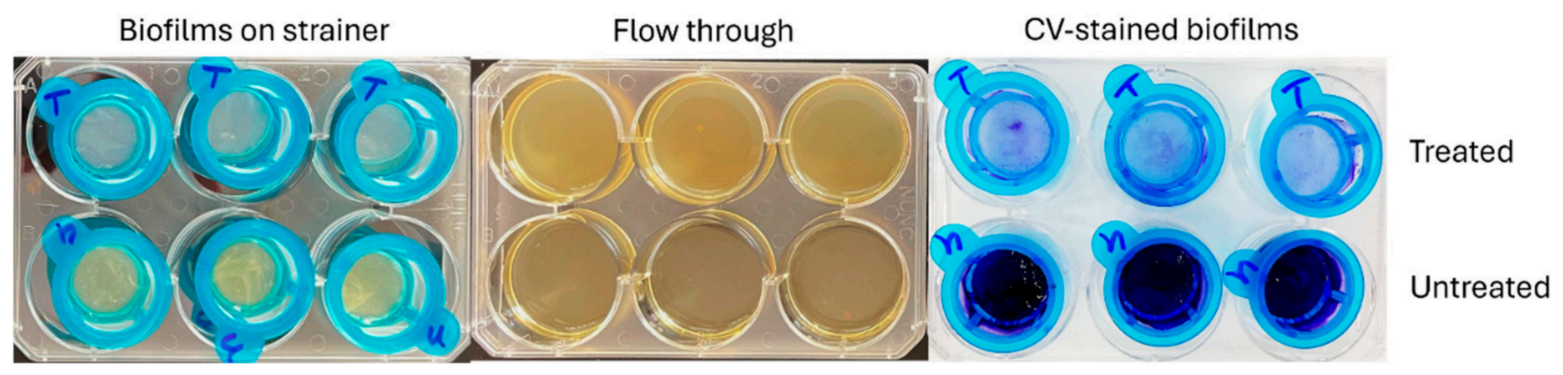
CAase-treated and untreated *C. jejuni* biofilms. Representative images of biofilms formed by *C. jejuni* 81-176 following treatment with 0.1 mg/mL CAase (**upper row**) compared with untreated controls (**lower row**). Biofilms were collected using nylon strainers. The left panel shows intact biofilms retained on the strainer surface, the middle panel displays the flow-through fractions collected beneath the strainer, and the right panel presents crystal violet–stained biofilms used for visualization and biomass quantification. Each treatment was performed in triplicate across three independent biological replicates.

CAase treatment significantly reduced biofilm biomass in both strains and under all growth setups. Representative images in Figure 2 show robust biofilms formed by *C. jejuni* 81-176 retained on 40 µm cell strainers. Following CAase exposure, the biofilms were visibly disrupted and released through the strainer, resulting in increased turbidity in the flow-through (Figure 2).

**Figure 3:**
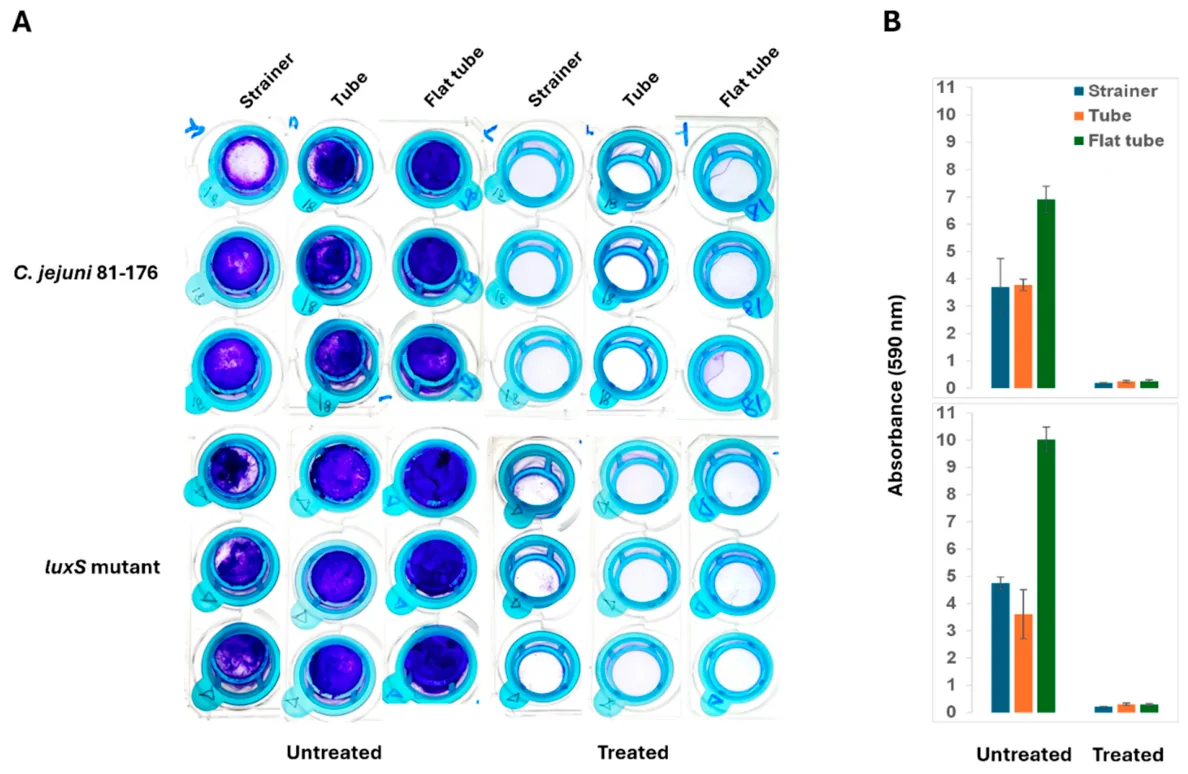
CAase-treated and untreated biofilms of *C. jejuni* 81-176 and its *luxS* mutant. (**A**) Biofilms were developed using three growth setups: a 14 mL round-bottom tube, a 10-cm^2^ flat-surface tissue culture tube, and a 40 µm cell strainer. (**B**) Biofilm biomass was quantified by crystal violet staining followed by OD590 measurement. Statistical differences between CAase-treated and untreated samples were analyzed using the Tukey–Kramer test (α = 0.05). Each sample included three biological replicates and two technical replicates.

Quantitative crystal violet assays showed that treatment with 0.1 mg/mL CAase at 42 °C for 2 h reduced biofilm biomass in *C. jejuni* 81-176 by more than 93%. The *luxS* mutant, despite its altered EPS composition resulting from disrupted quorum sensing, remained highly susceptible, exhibiting over a 91% reduction in biomass (Figure 3). These findings indicate that CAase effectively removes mature biofilms regardless of genetic or structural variation. Overall, the results here suggest that CAase functions as a potent biofilm-disrupting enzyme capable of degrading biofilms across *C. jejuni* strains with distinct EPS backgrounds.

### 3.3. SEM Examination of C. jejuni Biofilms

**Figure 4:**
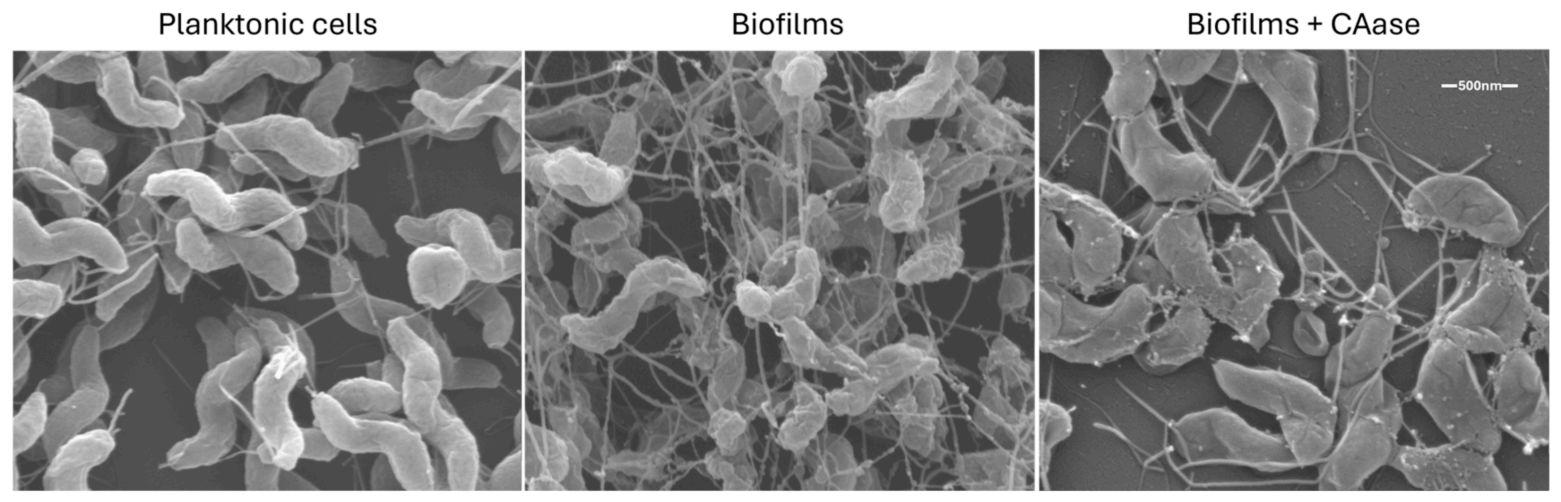
Scanning electron microscopy images of *C. jejuni* 81-176 biofilms under CAase-treated and untreated conditions. A planktonic cell control, lacking biofilm and extensive EPS, was included and processed identically. The images depict differences in cell morphology, surface attachment, and extracellular matrix architecture following 0.1 mg/mL CAase treatment, illustrating the potential structural effects of CAase on *Campylobacter* biofilms.

SEM imaging was used to evaluate the effects of CAase on *C. jejuni* 81-176 cell morphology and biofilm structure. As a control, planktonic cells lacking biofilm and extensive EPS displayed the typical spiral morphology of *Campylobacter* (Figure 4). In contrast, mature biofilms exhibited abundant EPS and a more three-dimensional architecture, with spiral cells embedded within a dense matrix.

CAase-treated biofilms exhibited clear structural deterioration. SEM imaging showed a marked reduction in extracellular matrix material and noticeable changes in cell morphology, indicating compromised matrix integrity and diminished overall biofilm stability. These observations collectively highlight the disruptive impact of CAase on *C. jejuni* biofilms.

### 3.4. Effect of CAase on C. jejuni Biofilm Kinematic Viscosity

**Figure 5:**
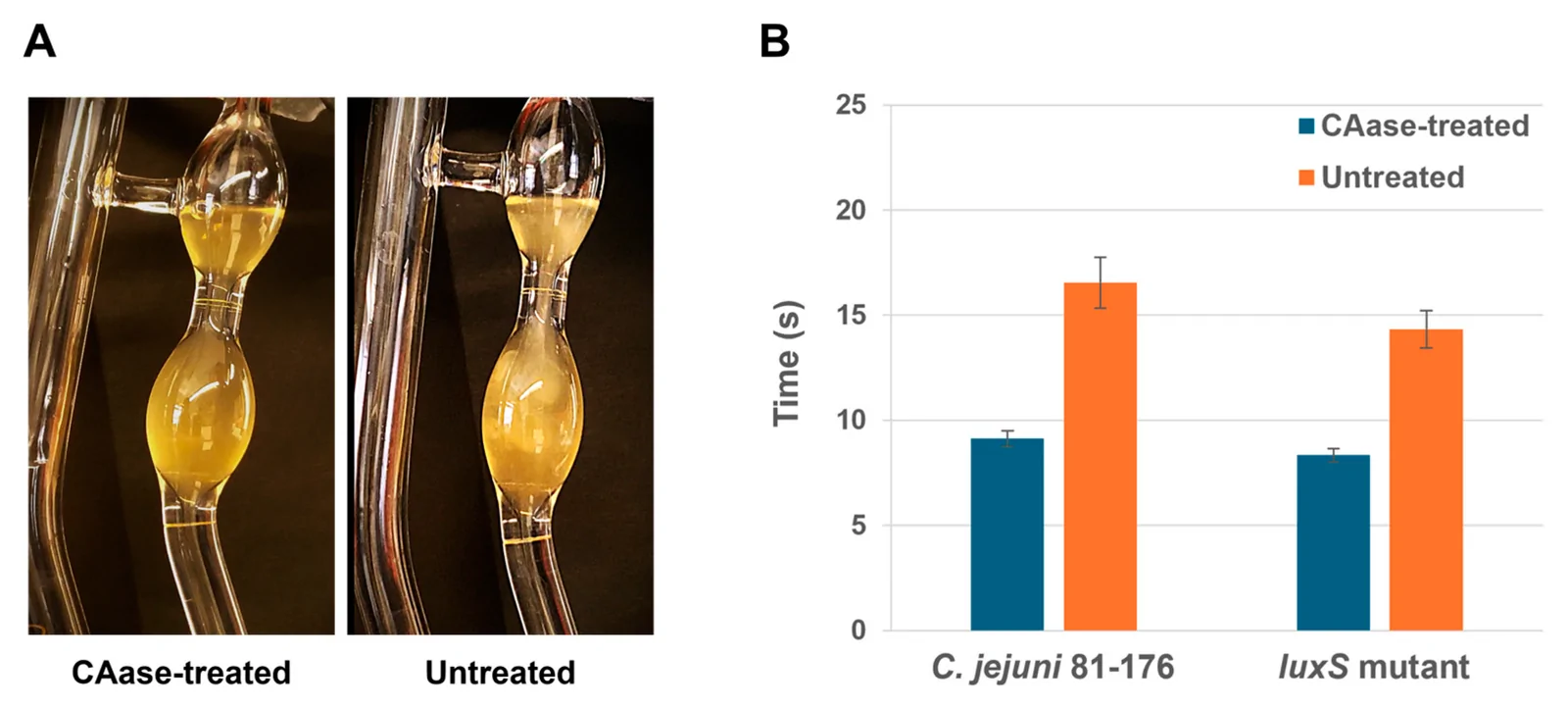
Kinematic viscosity of *C. jejuni* biofilms treated with and without CAase. (**A**) Flow times of the biofilm samples were measured using a glass kinematic viscometer tube to compare rheological properties following enzymatic degradation. (**B**) CAase treatment significantly decreased flow time for both *C. jejuni* 81-176 and the *luxS* mutant, reducing flow times from 16.5 s to 9.1 s and from 14.3 s to 8.3 s, respectively (*p* < 0.05, one-way ANOVA). Error bars represent the mean ± standard error of the mean (SEM) from three biological replicates.

To assess changes in mechanical properties, the flow behavior of *Campylobacter* biofilms with and without CAase treatment was evaluated by measuring kinematic viscosity. As shown in Figure 5 and Supplemental Video S1 (untreated) and Video S2 (treated), CAase significantly altered the rheological properties of both *C. jejuni* 81-176 and its *luxS* mutant. CAase-treated samples appeared more homogeneous and exhibited a marked reduction in “gelatinous” biomass. Flow time decreased from 16.5 to 9.1 s (corresponding to a viscosity reduction from 1.65 to 0.91 cSt) for the wild type and from 14.3 to 8.3 s (1.43 to 0.83 cSt) for the *luxS* mutant (*p* < 0.05, one-way ANOVA). These reductions in viscosity are consistent with CAase-mediated degradation of the biofilm matrix. This reduction reflects decreased biofilm viscosity consistent with CAase-mediated matrix disruption.

Lower viscosity reflects a weakened, less cohesive biofilm structure, likely increasing susceptibility to shear forces and promoting cell dispersion. This mechanical destabilization may also enhance penetration of antimicrobials or host defenses, ultimately reducing biofilm persistence under environmental or processing conditions.

## 4. Discussion

CAase treatment resulted in pronounced structural degradation of *C. jejuni* biofilms. SEM imaging revealed a clear reduction in extracellular matrix material, disrupted surface architecture, and altered cell morphology, indicating substantial loss of matrix integrity and overall biofilm stability. These structural changes were consistent with the marked reductions in biomass observed in crystal-violet assays and reinforce the conclusion that CAase effectively destabilizes the biofilm scaffold.

Because crystal violet staining and SEM do not differentiate viable from non-viable cells, we evaluated cell survival using CFU enumeration following CAase treatment. These results confirmed that CAase did not affect cell viability, indicating that the enzyme disrupts the biofilm matrix without killing the bacteria. This distinction is now explicitly acknowledged, clarifying that CAase’s activity reflects structural, not bactericidal, effects.

The viscosity measurements provide additional but indirect evidence for CAase-mediated weakening of the biofilm matrix. Both wild-type and *luxS*-mutant exhibited reduced flow time and kinematic viscosity following CAase treatment, suggesting loss of mechanical cohesion. These rheological changes may arise from multiple factors, including EPS degradation, decreased biomass, altered aggregation, and disruption of three-dimensional architecture, and therefore should not be interpreted as direct evidence of glycan hydrolysis. Instead, they support the broader conclusion that CAase compromises the biofilm’s mechanical integrity.

Importantly, CAase did not inhibit planktonic growth, underscoring that its activity is directed toward extracellular matrix components rather than cellular metabolism. Its effectiveness in both wild-type and *luxS*-mutant strains indicates that CAase can disrupt biofilms across distinct EPS backgrounds. While this consistency suggests possible interaction with glycan elements important for biofilm stability, such mechanistic interpretations remain inferential, as EPS composition and glycan degradation products were not directly measured.

The effects of CAase align with those of other glycosyl hydrolases that disrupt biofilms by cleaving matrix polymers. DNase I, for example, degrades extracellular DNA and enhances dispersal in *C. jejuni* and multispecies biofilms [31]. while cellulase and α-amylase disperse *Pseudomonas aeruginosa* and *Staphylococcus aureus* biofilms in a context-dependent manner [25]. GHs from *Pseudomonas fluorescens* also efficiently dismantle polysaccharide-rich biofilms at low concentrations [32]. Within this framework, CAase exhibits comparable biofilm-disrupting activity and shows promise as part of enzyme-based biofilm-control strategies.

Together, the structural, rheological, and quantitative data demonstrate that CAase compromises multiple aspects of *C. jejuni* biofilm integrity, biochemically by altering matrix composition and mechanically by reducing cohesive strength, while leaving cell viability unaffected. These findings support CAase as a promising antibiofilm candidate that may enhance sanitation effectiveness by improving antimicrobial penetration and reducing reliance on harsh chemical treatments. Its consistent activity across genetically distinct *C. jejuni* strains, as well as other foodborne pathogens such as *Escherichia coli* O157:H7, *Salmonella enterica* serotype Typhimurium, and *Listeria monocytogenes* [27], further underscores its potential utility in diverse industrial and environmental settings.

However, the present study was conducted using laboratory-grown, pure-culture *C. jejuni* biofilms under controlled conditions. Mixed-species biofilms typical of food-processing environments were not evaluated, and the enzyme’s stability across fluctuating temperatures, pH conditions, oxidizing sanitizers, detergents, or organic residues remains unknown. Industrial applicability is therefore prospective. Future studies should assess CAase stability and performance under processing-relevant conditions, evaluate its compatibility with commonly used sanitizers, and examine its activity on biofilms formed on industry-relevant materials. Expanding these analyses to additional foodborne and environmental pathogens will further clarify the enzyme’s practical utility and inform its integration into real-world biofilm-control interventions.

## 5. Conclusions

This study demonstrates that although CAase does not inhibit planktonic growth of *C. jejuni*, it exerts a strong disruptive effect on established biofilms by degrading key extracellular matrix components. CAase treatment substantially reduced biofilm biomass, weakened structural cohesion, altered rheological properties, and produced marked architectural damage observable by SEM. Its consistent activity against both wild-type and *luxS*-mutant strains suggests that CAase may act on glycan elements important for biofilm stability, although this remains a hypothesis rather than a mechanism directly demonstrated here. Collectively, these findings support CAase as a promising antibiofilm candidate for disrupting persistent *C. jejuni* biofilms. Further mechanistic and applied studies will be necessary to define its full utility and evaluate its effectiveness under broader environmental and industrial conditions.

## Data Availability

The original contributions presented in this study are included in the article/Supplementary Materials. Further inquiries can be directed to the corresponding author.

## Supplementary Materials

The following supporting information can be downloaded at https://www.mdpi.com/article/10.3390/foods15162849/s1. Supplemental Video S1. Kinematic viscosity of untreated *C. jejuni* 81-176 biofilms was measured using a glass kinematic viscometer tube; Supplemental Video S2. Kinematic viscosity of CAase-treated *C. jejuni* 81-176 biofilms was measured using a glass kinematic viscometer tube.
